# A Three Stage Integrative Pathway Search (*TIPS*^©^) framework to identify toxicity relevant genes and pathways

**DOI:** 10.1186/1471-2105-8-202

**Published:** 2007-06-14

**Authors:** Zheng Li, Shireesh Srivastava, Sheenu Mittal, Xuerui Yang, Lufang Sheng, Christina Chan

**Affiliations:** 1 Department of Chemical Engineering and Materials Science, Michigan State University, East Lansing, MI 48824, USA; 2 Department of Biochemistry and Molecular Biology, Michigan State University, East Lansing, MI 48824, USA; 3 Department of Computer Science and Engineering, Michigan State University, East Lansing, MI 48824, USA; 4 MetagenX LLC. Okemos, MI, 48864, USA; 5 Department of Biochemistry and Molecular Biology, Michigan State University, East Lansing, MI 48824, USA; 6 National Institute on Alcohol Abuse and Alcoholism (NIAAA/NIH), 5625 Fishers Lane, Rockville, MD 20851, USA

## Abstract

**Background:**

The ability to obtain profiles of gene expressions, proteins and metabolites with the advent of high throughput technologies has advanced the study of pathway and network reconstruction. Genome-wide network reconstruction requires either interaction measurements or large amount of perturbation data, often not available for mammalian cell systems. To overcome these shortcomings, we developed a Three Stage Integrative Pathway Search (*TIPS*^©^) approach to reconstruct context-specific active pathways involved in conferring a specific phenotype, from limited amount of perturbation data. The approach was tested on human liver cells to identify pathways that confer cytotoxicity.

**Results:**

This paper presents a systems approach that integrates gene expression and cytotoxicity profiles to identify a network of pathways involved in free fatty acid (FFA) and tumor necrosis factor-α (TNF-α) induced cytotoxicity in human hepatoblastoma cells (HepG2/C3A). Cytotoxicity relevant genes were first identified and then used to reconstruct a network using Bayesian network (BN) analysis. BN inference was used subsequently to predict the effects of perturbing a gene on the other genes in the network and on the cytotoxicity. These predictions were subsequently confirmed through the published literature and further experiments.

**Conclusion:**

The *TIPS*^© ^approach is able to reconstruct active pathways that confer a particular phenotype by integrating gene expression and phenotypic profiles. A web-based version of *TIPS*^© ^that performs the analysis described herein can be accessed at .

## Background

The regulation of cellular functions is achieved through the contribution and interactions of genetic, signaling and metabolic pathways. Consequently, cellular processes may be singularly regulated, i.e., at the gene or transcription level, or controlled by a network of interactions of genes, proteins and metabolites. Therefore, understanding the biological function of the myriad of genes and how these genes and gene products interact and regulate each other to yield a functional cell would help to identify more appropriate pathways that should be targeted or studied for a given disease.

Novel analytical frameworks are required to unravel the regulatory and functional relationships from profiles of genes, proteins and metabolites. Approaches have been developed to infer genome wide networks from high throughput data. These approaches typically require genome-wide interaction data [1,2] or a wide range of perturbation data [3-5], which have been applied to yeast and human B cells, respectively, to reverse engineer genome wide gene regulatory networks. Unfortunately, genome-wide interaction measurements as well as large amounts of perturbation data are not easily or readily obtainable for researchers working with mammalian systems. Thus, a challenge remains to reconstruct networks from a small amount of perturbation data. The small perturbation data size poses a challenge to statistical inference and makes it extremely hard to infer genome wide networks with any degree of confidence [6-8]. To address this, we developed an approach to identify a smaller network of active pathways rather than genome wide networks based upon a subset of important genes selected by integrating gene expression and phenotypic profiles. The approach first integrates genetic algorithm coupled partial least squares analysis (GA/PLS) [9] and constrained independent component analysis (CICA) [10] to identify a subset of genes relevant to a phenotype. Next, BN analysis is used to reconstruct an active sub-network from this smaller group of genes to reveal which pathways are induced by the external stimuli or environmental factors. Applying BN to a reduced subset of genes also circumvents the inefficiency of the BN analysis in inferring large size networks, such as is the case with genome wide networks. BN can detect indirect influences and unmeasured events and is not susceptible to the existence of unobserved variables [6]. It has been applied to infer gene regulatory network of yeast cell cycle from gene expression data, metabolic sub-networks from metabolic data and protein signaling network from protein activity data [6,11-13]. The reconstructed network was then used to predict the effect of perturbing a gene on the other genes in the network with BN inference, and thus provided insight into how the genes interacted within the network to produce a specific phenotype.

As proof-of-concept, the framework was applied to identify the pathways that confer cytotoxicity in HepG2/C3A cells. Liver toxicity is often used to assess the safety of drugs and is a primary reason for drug recalls [14]. Therefore, predicting liver toxicity earlier in the drug development process would be valuable. In the current study, saturated FFA, palmitate, was found to induce liver toxicity, and this effect was exacerbated by the presence of TNF-α. Indeed, elevated levels of FFAs and TNF-α have been shown to be involved in the pathogenesis of liver disorders, such as fatty liver disease and steatohepatitis [15-18]. Therefore, we applied our approach to this model system from the standpoint that if we can identify pathways that may attenuate the toxicity in the presence of FFAs and TNF-α, perhaps this model could be applied eventually to drug candidates to identify pathways that may be modulated to enhance the efficacy and minimize the toxicity of the drug. We analyzed the genetic responses of the hepatocytes to physiologically elevated levels of FFAs and TNF-α to identify pathways involved in conferring cytotoxicity, and which in turn may provide insight into the physiological actions of these factors.

We developed a *TIPS*^© ^framework that first applied GA/PLS [9] and independent component analysis (ICA) [19,20] to identify a subset of genes relevant to cytotoxicity. We assumed, as a first approximation, a log linear relationship between gene expression and cytotoxicity. The genes selected by GA/PLS were initially corroborated with published results to identify known interactions. In order to extract an independent pathway related to a phenotype, such as cytotoxicity, from the gene expression profile, we propose a constrained ICA (CICA) approach. The relevance of the genes to the toxicity identified by GA/PLS along with the cytotoxicity profiles were used as constraints in CICA. CICA extracted a phenotype-relevant-component from the gene expression data. CICA assumes that the expression profile of thousands of genes can be represented by a reduced number of mutually independent processes. Biologically meaningful gene groups have been successfully identified by ICA [19,20]. A phenotype-relevant-component was identified by minimizing the mutual information between the phenotype-relevant-component and the other independent components while maximizing the correlation between the component and the constraints. The expression profiles of the genes with the highest weights in CICA were used in BN analysis for network reconstruction. The reconstructed network was perturbed to identify i) which genes, when perturbed, had an impact on altering the cytotoxic phenotype in the palmitate cultures, and ii) how perturbing one gene (node) affected the other genes in the network. The reconstructed network provided potential explanation(s) on how palmitate and TNF-α induced cytotoxicity. The model identified (i) the roles played by stearoyl-CoA desaturase (SCD), double-stranded-RNA-dependent protein kinase (PKR) and Bcl-2 in the palmitate-induced cytotoxicity, and (ii) the activation of nuclear factor kappa B (NF-κB) by TNF-α is mediated by protein kinase C delta (PKC-δ). These simulated perturbations of the reconstructed network were evaluated experimentally to assess the accuracy of the predictions.

## Results

### 1. Identifying genes relevant to cytotoxicity using GA/PLS

**Figure 1:**
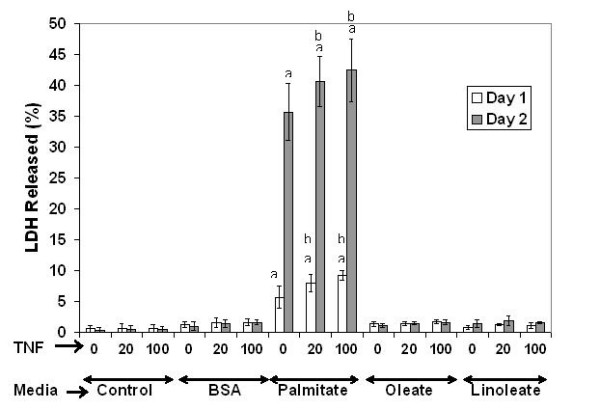
**Cytotoxicity (LDH release)**. Confluent HepG2 cells exposed to different types of fatty acids (0.7 mM, complexed to 4% w/v BSA) and TNF-α for 24 and 48 hours. X-axis labels indicate the TNF-α concentration in ng/ml and the medium employed in each condition. Data expressed as averages of nine samples +/- s.d. from three independent experiments. **a**, Significant medium effect, P < 0.05 relative to control (BSA medium with no TNF-α); **b**, Significant TNF-α effect within a treatment, P < 0.05 compared to corresponding medium with no TNF-α exposure. TNF-α concentrations are in ng/ml.

**Figure 2:**
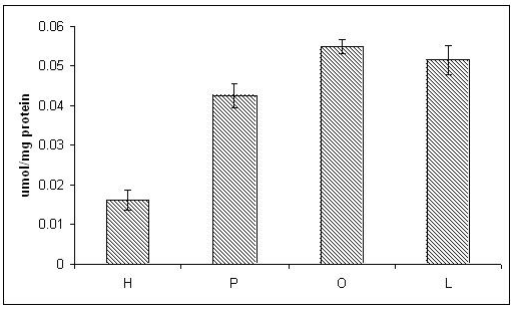
**Intracellular TG accumulation**. Intracellular TG accumulation increased in FFA treated cells. * significantly higher than control, p < 0.01 by t-test. H: control, P: palmitate treatment, O: oleate treatment, L: linoleate treatment.

Lactate dehydrogenase (LDH) release was measured as an indicator of the cytotoxicity. We found that exposing HepG2/C3A cells to FFAs (palmitate, oleate, or linoleate) in the presence and absence of TNF-α, only palmitate was cytotoxic to the cells and resulted in significantly higher LDH release (Figure 1). TNF-α alone was not toxic to the cells. The cytotoxic effect of TNF-α was observed only in the palmitate-treated cells. Exposure to oleate or linoleate was not cytotoxic, but caused the cells to accumulate intracellular triglycerides (Figure 2). To obtain a global view of the changes induced by FFAs and TNF-α, we capitalized upon high-throughput cDNA microarrays to quantify the gene expression profiles of the HepG2/C3A cells.

**Figure 3:**
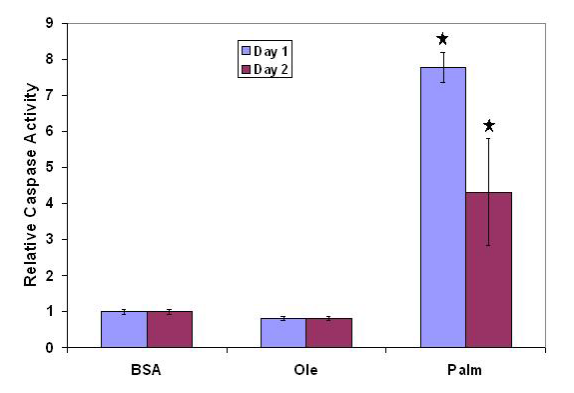
**Caspase activity**. Palmitate treatment increased caspase-3 activity significantly as compared to control (BSA) and unsaturated fatty acid (oleate). * significantly higher in palmitate, p < 0.01 by t-test. BSA: control, Ole: oleate treatment, Palm: palmitate treatment.

We applied GA/PLS to the gene expression and LDH release profiles. The GA/PLS algorithm counted the frequency with which each gene was selected to predict LDH release and provided a measure of the relevance of each gene to LDH release [9]. The genes with high frequency were organized into functional groups based on the literature information on the functional role of these genes, as shown in Table 1. Evaluating the groups of genes assigned high frequency by GA/PLS suggested that the functional groups such as oxidative stress, apoptosis, TNF-signaling, mitochondria were relevant to the cytotoxicity. Several of these potential mechanisms suggested by the GA/PLS results were experimentally validated. For example, the identification of oxidative stress related genes suggested a possible involvement of reactive oxygen species (ROS) in the palmitate-induced cytotoxicity. Indeed, we found the ROS level was elevated in the palmitate cultured cells and adding ROS scavengers prevented the cytotoxicity induced by palmitate [21]. In addition, the caspase-3 activities were significantly elevated in the palmitate cultures as shown in Figure 3, which corroborated the involvement of apoptosis in the observed cytotoxicity. Finally, identification of translocase of outer/inner mitochondria membrane (TOM/TIM) which are known to be related to mitochondrial potential [22] were corroborated by the reduced mitochondrial potential in the palmitate cultured cells [21].

**Table 1:** Functional groups of genes related to LDH release, selected by GA/PLS

**Functional Category**	**Accession Number**	**Gene name**	**Frequency**
**Oxidative Stress**			
	**AA418907**	(gC) cytochrome P450, polypeptide 1 (CYP1A1)	12
	**H93482**	(g) glutamate-cysteine ligase, catalytic subunit	11
	**AA111999**	(gC) NADH dehydrogenase (ubiquinone) 1 alpha subcomplex, 1	10
	**T73468**	(gC) glutathione S-transferase A2 (GSTA2)	10
	**T73294**	(gC) P450 (cytochrome) oxidoreductase (POR)	10
	**H99813**	(gC) glutathione S-transferase theta 1 (GSTT1)	10
	**AA460251**	(gC) NADH dehydrogenase (ubiquinone) 1	9
	**AA046701**	(gC) ATP synthase, H+ transporting, subunit c	8
	**AA291163**	(gC) glutaredoxin (thioltransferase) (GLRX)	8
	**AA777289**	(gC) glutathione reductase (GSR)	8
	**R63065**	(gC) glutathione S-transferase M3	8
	**AA664180**	(gC) glutathione peroxidase 3 (plasma) (GPX3)	7
	**H53340**	(gC) metallothionein 1G (MT1G)	7
	**N72263**	(gC) NADH dehydrogenase (ubiquinone) 1 alpha subcomplex, 10	7
	**AA865265**	(gC) cytochrome c (HCS)	7
	**N21576**	(gC) cytochrome P450, subfamily XXIV (CYP24)	7
	**AA022627**	(gC) NADH dehydrogenase (ubiquinone) 1 alpha subcomplex, 7	7
	**T72259**	(gK) cytochrome P450, polypeptide 7 (CYP2A7)	7
	**AA281549**	(gM) holocytochrome c synthase (HCCS)	7
	**AA490938**	(gM) NAD(P)H dehydrogenase, quinone 2 (NQO2), mRNA	7
	**W73474**	(gC) microsomal glutathione S-transferase 2 (MGST2)	7
	**T98002**	(gC) cytochrome P450, subfamily IVF, polypeptide 12 (CYP4F12)	7
**TCA Cycle**			
	**AA679907**	(gC) isocitrate dehydrogenase 2 (NADP+), mitochondrial (IDH2)	12
	**N67639**	(gC) citrate synthase (CS)	8
	**AA234519**	(gC) succinate-CoA ligase, GDP-forming, beta subunit	7
**Transcription factor**			
	**AA406269**	(g) nuclear factor I/X (CCAAT-binding transcription factor) (NFIX)	12
	**AA24743**	(gC) zinc finger protein 36, C3H type-like 1 (ZFP36L1)	9
	**AA608536**	(gC) inhibitor of kappa light polypeptide gene enhancer in B-cells	7
**TNF- Signal**			
	**AA134814**	(gC) TRAF family member-associated NFκβ activator (TANK)	9
	**W72329**	(gC) lymphotoxin alpha (TNF- superfamily, member 1) (LTA)	9
	**AA486789**	(gC) Fas (TNF-RSF6) associated factor 1 (FAF1)	8
	**AA443577**	(gC) tumor necrosis factor (ligand) superfamily, member 13 (TNF-SF13)	8
	**AA625666**	(gC) LPS-induced TNF-alpha factor (PIG7)	8
	**AA433944**	(gC) Fas (TNF-RSF6)-associated via death domain (FADD)	7
	**AA476272**	(g) tumor necrosis factor, alpha-induced protein 3 (TNF-AIP3)	7
	**H98636**	(gK) tumor necrosis factor receptor superfamily, member 5 (TNF-RSF5)	7
	**N50859**	(gC) TNF-AIP3 interacting protein 2 (TNIP2)	7
	**T64483**	(gC) TNF-AIP3 interacting protein 1 (TNIP1)	7
	**R71691**	(gC) TNF- receptor-associated factor 1 (TRAF1)	7
	**AA456314**	(gC) tumor necrosis factor, alpha-induced protein 1 (TNF-AIP1)	7
	**AA778663**	(gC) tumor necrosis factor (ligand) superfamily, member 9 (TNF-SF9)	7
**Signal pathways**			
	**R32848**	(gC) S100 calcium binding protein P (S100P)	12
	**T62952**	(gC) protein phosphatase 4, regulatory subunit 1 (PPP4R1)	10
	**AA019459**	(gC) protein tyrosine kinase 9 (PTK9)	10
	**R72296**	(gC) protein phosphatase 1, regulatory (inhibitor) subunit 15B	10
	**AA598996**	(gM) solute carrier family 38, member 2 (SLC38A2)	10
	**AA496810**	(gM) protein kinase C substrate 80K-H (PRKCSH)	10
	**AA454810**	(g) tumor-associated calcium signal transducer 2 (TACSTD2)	10
	**AA453754**	(gC) serine/threonine kinase 17a (apoptosis-inducing) (STK17A)	9
	**AA488413**	(gC) MAP kinase-interacting serine/threonine kinase 2 (MKNK2)	9
	**N47552**	(gC) mitogen-activated protein kinase 6	9
	**W68184**	(gC) phospholipase A2, group IVB (cytosolic)	9
	**AA233185**	(gC) insulin-like growth factor binding protein 1 (IGFBP1)	9
	**R45941**	(gM) protein tyrosine phosphatase, receptor type, N (PTPRN)	8
	**W79920**	(gN) G protein-coupled receptor 87 (GPR87)	8
	**H01164**	(gC) serine/threonine kinase 17a (apoptosis-inducing) (STK17A)	8
	**AA45819**	(gC) mitogen-activated protein kinase 3	8
	**AA678095**	(gM) G protein-coupled receptor 48 (GPR48)	8
	**AA487028**	(gM) protein phosphatase 1, regulatory (inhibitor) subunit 12A (PPP1R12A)	8
	**H90855**	(gC) mitogen-activated protein kinase kinase kinase 7	8
	**AA485347**	(gM) protein phosphatase 1, regulatory (inhibitor) subunit 11 (PPP1R11)	8
	**AA789328**	(gC) cyclin-dependent kinase (CDC2-like) 10 (CDK10)	8
	**W44762**	(gC) potassium inwardly-rectifying channel, subfamily J, member 2 (KCNJ2)	8
	**R44740**	(gC) mitogen-activated protein kinase kinase 1 (MAP2K1)	8
	**AA047570**	(gM) phospholipase C, delta 4 (PLCD4)	7
	**H11054**	(gC) protein kinase C, delta	7
	**AA443982**	(gC) protein phosphatase 1, catalytic subunit, alpha isoform (PPP1CA)	7
	**AA480906**	(gC) protein kinase C binding protein 1	7
	**AA490473**	(gC) protein phosphatase 2 (formerly 2A), catalytic subunit	7
	**AA679208**	(gC) MAP3K7IP1	7
	**R19158**	(gC) serine/threonine kinase 6 (STK6)	7
	**R79082**	(gC) protein tyrosine phosphatase, receptor type, K (PTPRK)	7
	**R50953**	(gC) MAP4K2	7
	**AA862435**	(gC) G protein-coupled receptor kinase 5 (GPRK5)	7
	**AA464590**	(gC) protein tyrosine phosphatase, receptor type, N polypeptide 2 (PTPRN2)	7
**Fatty acid metabolism**			
	**AA877618**	(gC) fatty acid amide hydrolase (FAAH)	12
	**H29215**	(gC) fatty-acid-Coenzyme A ligase, long-chain 3 (FACL3)	9
	**T98355**	(gC) long-chain fatty-acyl elongase (LCE)	7
	**AA678178**	(gC) fatty acid amide hydrolase	7
**Apoptosis**			
	**AA446839**	(gC) BCL2/adenovirus E1B 19kDa interacting protein 3 (BNIP3)	10
	**W45688**	(gN) caspase 6, apoptosis-related cysteine protease (CASP6)	10
	**AA676836**	(gC) acid sphingomyelinase-like phosphodiesterase (ASM3A)	9
	**AA448468**	(gC) caspase 8, apoptosis-related cysteine protease (CASP8)	8
	**H80712**	(gC) caspase 10, apoptosis-related cysteine protease (CASP10)	8
	**AA496782**	(gF) requiem, apoptosis response zinc finger gene (REQ)	8
	**AA165628**	(gC) UDP-glucose ceramide glucosyltransferase	7
	**AA630354**	(gC) sphingosine kinase 2 (SPHK2)	7
	**AA156940**	(gC) programmed cell death 5 (PDCD5)	7
	**AA459381**	(gC) sphingosine-1-phosphate lyase 1	7
	**T57777**	(gC) BCL2-like 13 (apoptosis facilitator) (BCL2L13)	7
**Translation**			
	**AA456664**	(gM) eukaryotic translation termination factor 1 (ETF1)	10
	**AA027240**	(gC) eukaryotic translation initiation factor 2, subunit 2 beta, 38kDa (EIF2S2)	10
	**N72715**	(gC) translational inhibitor protein p14.5 (UK114)	8
	**AA916914**	(gC) eukaryotic translation initiation factor 3, subunit 10 theta	8
	**AA191463**	(gC) eukaryotic translation initiation factor 4 gamma, 3 (EIF4G3)	7
	**H92556**	(gC) eukaryotic translation initiation factor 2B, subunit 2 beta, 39kDa (EIF2B2)	8
**Mitochondria Related**			
	**AA457118**	(gC) translocase of outer mitochondrial membrane 34 (TOMM34)	10
	**R86713**	(gC) translocase of inner mitochondrial membrane 22 homolog (yeast) (TIMM22)	9
	**AA644550**	(gM) translocase of outer mitochondrial membrane 20	7

### 2. Identifying genes involved in an independent pathway related to cytotoxicity using CICA

GA/PLS determined the frequency with which each gene was selected to predict LDH release. The frequencies and the profile of LDH release were applied as structure and profile constraints respectively in CICA to extract a phenotype-relevant-component from the gene expression profile, see methods for more details. The independent component in this case identified a subset of genes whose profiles corresponded to the profile of LDH release. CICA determined the weights for each gene by minimizing the mutual information between the independent components and maximizing the correlation to the constraints. The weights determined by CICA are in the additional files [see Additional file 1]. Genes that had weights significantly different from zero with a 95% confidence using the Z-test were subjected to BN analysis for pathway reconstruction.

### 3. Reconstruct pathways related to cytotoxicity using BN

**Figure 4:**
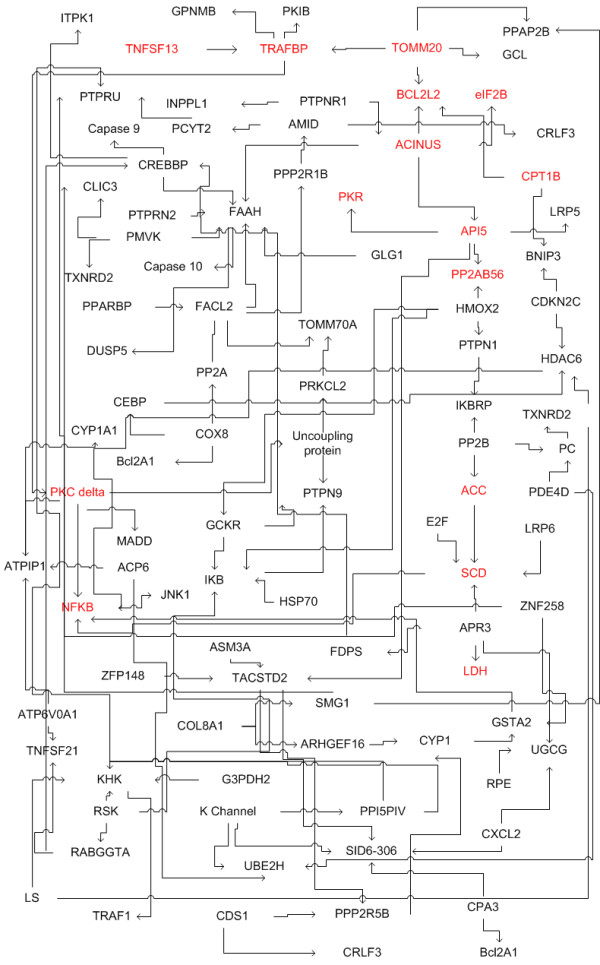
**Network reconstructed with constraints based algorithm**. GA/PLS and ICA selected the relevant genes, and BN analysis reconstructed the network using the selected subset of genes. The network provides an overview of the factors and pathways involved in regulating cytotoxicity. The nodes discussed in the paper are highlighted in red. Microsoft Visio was used to generate the Figure.

BN reconstructed how the genes, identified by CICA, are connected in a network and involved in regulating cytotoxicity. The resulting network is shown in Figure 4. To evaluate potential pathways involved in palmitate-induced cytotoxicity, we performed *in silico *perturbation analyses with BN inference and experimentally validated the reconstructed network.

#### 3.1 Perturbation of the reconstructed network

The reconstructed network allowed us to perturb the network *in silico *to identify which nodes had an impact on modulating LDH release. BN inference was used to predict the effect of perturbing a single gene/node on i) the other gene within the network, and ii) the level of LDH release in the palmitate cultures. The predictions of the simulated perturbations were subsequently confirmed with inhibitor (or activator) experiments. We altered the activity of the nodes by inhibiting (activating) the protein activity. Perturbations of the SCD (see section 3.2) and PKR (section 3.3) were simulated with BN inference to evaluate their effects on cytotoxicity, whereas PKC-δ (section 3.4) was perturbed to predict its effect on NF-κB activation.

#### 3.2 Role of stearoyl-CoA desaturase (SCD) in palmitate-induced cytotoxicity

**Figure 5:**
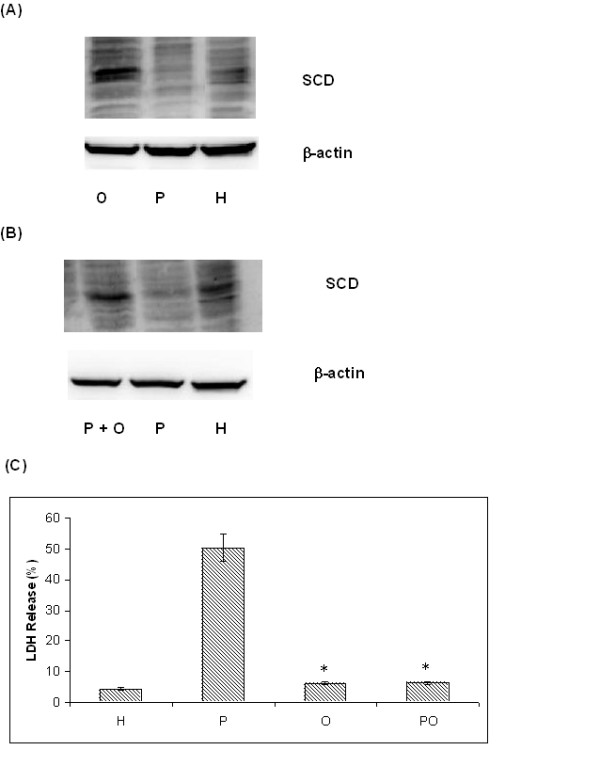
**Effect of palmitate on stearoyl-CoA desaturase (SCD) measured by western blotting**. (A) SCD was downregulated in the palmitate (0.7 mM) cultures as compared to the oleate (0.7 mM) and control cultures. (B) Co-supplementation of oleate (0.3 mM) with palmitate (0.4 mM) prevented the downregulation of SCD. (C) Co-supplementing palmitate (0.4 mM) with oleate (0.3 mM) decreased LDH release significantly, P < 0.01 (t-test). P: treated with 0.7 mM palmitate for 48 hours, PO: treated with 0.4 mM palmitate plus 0.3 mM oleate for 48 hours. Data expressed as average +/- SD from three independent experiments, * significantly lower than palmitate, p < 0.01 by t-test.

SCD was found closely connected to LDH and acetyl-CoA carboxylase (ACC) in the reconstructed network. Both of these connections are supported by the literature [23]. SCD is the rate-limiting enzyme to produce monounsaturated fatty acids. Its deficiency has been found to increase fatty acid oxidation by activating AMP-activated protein kinase (AMPK) in the liver. AMPK phosphorylates ACC at Ser-79 which leads to the inhibition of ACC activity and decreased malonyl-CoA concentration [23]. Malonyl-CoA inhibits carnitine palmitoyl-CoA transferase (CPT-1) [24]. Thus, a decrease in the levels of malonyl-CoA activates CPT-1 and increases fatty acid beta-oxidation in the mitochondrion. In the palmitate cultures, the protein expression level of SCD (Figure 5A) was reduced as compared to the control and the oleate cultures. However, co-supplementing the palmitate cultures with oleate restored the SCD protein expression level (Figure 5B) and correspondingly reduced the LDH release (Figure 5C), which indicated a protective role of SCD. This may explain, in part, the preference of the HepG2/C3A cells to oxidize palmitate as opposed to synthesizing triglycerides from it, as was done with the unsaturated fatty acids. In support of this, knockout of the SCD gene in mice has been found to increase mitochondrial fatty acid oxidation [25].

To investigate the role of SCD in modulating palmitate-induced cytotoxicity, we simulated an upregulation of SCD in the reconstructed network by setting the SCD gene expression to a higher level *in silico*. Up-regulating SCD reduced the probability that the LDH release would remain high from ~67% to ~35% (Table 2). The simulation results agreed well with the literature, e.g., over-expressing SCD protected CHO cells from palmitate-induced cytotoxicity [26].

**Table 2:** Simulating genetic perturbation and its effects on LDH release

		**Probability of LDH Release**	
		
**Gene**	**Level**	**Low**	**High**
SCD	low	0.33	0.67
	high	0.65	0.35 ↓
NF-KB	low	0.47	0.53
	high	0.77	0.23↓
PKR	low	0.65	0.25↓
	high	0.57	0.43

The probability of LDH release taking on a high or low level in the palmitate cultures. BN inference was used to conduct the simulations of up-regulation of SCD, down-regulation of PKR, and up-regulation of NF-KB. "↓" indicates decreased probability and "↑" indicates increased probability.

**Figure 6:**
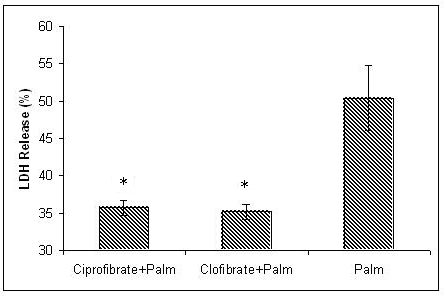
**Effect of SCD activators clofibrate and ciprofibrate on LDH release in the palmitate cultures**. Clofibrate and ciprofibrate are known to transcriptionally increase the activity of SCD. Palm: treated with 0.7 mM palmitate for 48 hours, Palm+clofibrate: treated with 50 μM clofibrate and 0.7 mM palmitate, Palm+ciprofibrate: treated with 50 μM ciprofibrate and 0.7 mM palmitate. 6 hours pretreatment followed by 48 hours co-supplementation of 50 μM of clofibrate or ciprofibrate significantly decrease LDH release in the palmitate culture, P < 0.01 (t-test). Data expressed as average +/- SD from three independent experiments, * significantly lower than in control, p < 0.01 by t-test. H: control, P: palmitate treatment, O: oleate treatment, P+O: palmitate and oleate co-supplementation.

To experimentally validate the simulation results, we supplemented the cell cultures with 50 μM of clofibrate or ciprofibrate to increase the SCD activity. The SCD1 activity can be transcriptionally activated by clofibrate or ciprofibrate, which are known to increase the activity of SCD through a PPAR independent pathway [27-29]. The fibrate supplementation significantly decreased the LDH release in the palmitate cultures (Figure 6). Therefore, BN inference correctly identified SCD as a relevant factor in palmitate-induced cytotoxicity.

#### 3.3 Role of Bcl-2 and PKR in palmitate-induced cytotoxicity

**Figure 7:**
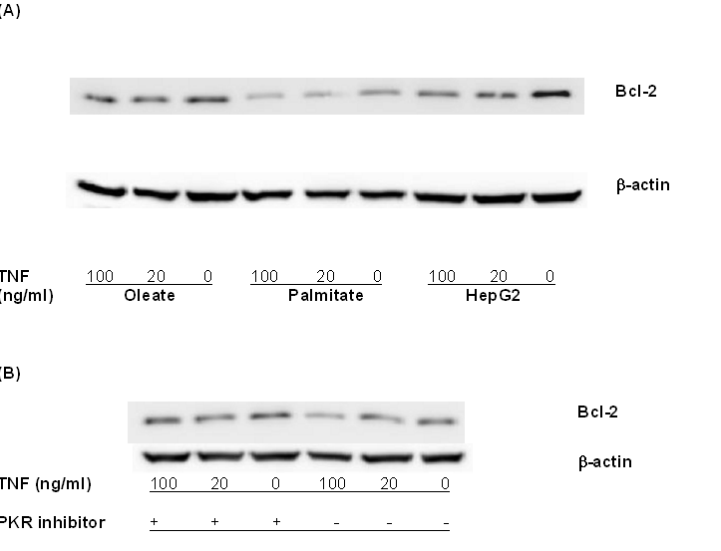
**Effect of palmitate and TNF-α on Bcl-2 expression measured by western blotting**. (A) TNF-α supplementation at 20–100 ng/ml downregulated Bcl-2 in the control, palmitate, and oleate cultures. Similarly, palmitate downregulated Bcl-2 protein expression level as compared to the control and oleate cultures. (B) Effect of PKR inhibition on Bcl-2 level in the palmitate cultures. PKR inhibitor (6 μM) increased the expression of Bcl-2 in palmitate cultured cells.

Bcl-2 is a group of proteins including pro-apoptotic members, such as Bax, Bid, Bad, and anti-apoptotic ones such as Bcl-2, Bcl-xl, Bcl-w. Anti-apoptotic Bcl-2 protein inhibits apoptosis by guarding the mitochondrial gate against the release of cytochrome c and the subsequent activation of caspases. Bcl-2 was found to be connected to factors such as PKR, TOM20, eIF2B and CPT-1 (Figure 4). The protein expression level of Bcl-2 in cultured HepG2 cells as a function of TNF-α concentrations was measured by western blotting (Figure 7A). TNF-α (20–100 ng/ml) suppressed the protein expression level of Bcl-2 in a dose-dependent manner. Palmitate, similarly, decreased the Bcl-2 protein expression level significantly as compared to the control and oleate cultures. The suppression of Bcl-2 may explain, in part, the cytotoxic effects of palmitate and TNF-α in the palmitate cultured cells (Figure 1). In support of this finding, over-expression of Bcl-2 in 2B4.11 T cell hybridoma cell lines have been shown to inhibit palmitate-induced cytotoxicity [30]. While TNF-α and oleate also reduced the Bcl-2 levels, they did not produce any overt toxicity by themselves. This indicates that perhaps, the reduction in Bcl-2 *alone *is not sufficient to *cause *toxicity. However, a reduction in Bcl-2 levels would *prime *the cells to other insults such as oxidative stress. Only palmitate, and not oleate or TNF-α, caused ROS production as well as a reduction in the Bcl-2 levels. Thus, reduced Bcl-2 levels would have only worsened the toxicity produced by the oxidative stress. Therefore, reducing Bcl-2 may be one of the ways in which FFA and TNF-α made cells susceptible to toxicity.

In the reconstructed network, Bcl-2 was connected to the translocase of outer membrane (TOM20) and carnitine palmitoyl transferase (CPT-1). These connections are supported by published literature results. Targeting the Bcl-2 protein to the mitochondria is mediated by the interaction between the C terminus of Bcl-2 and TOM20 [31]. Similarly, a direct interaction between Bcl-2 and CPT-1 has been confirmed through a co-immunoprecipitation study [32], thus a direct connection between Bcl-2 and CPT-1 found by the model is encouraging.

The model found PKR to be indirectly connected to Bcl-2. PKR, an interferon-inducible serine/threonine kinase, has been found to mediate a number of signal transduction pathways involved in immune response, tumorigenesis and regulation of apoptosis. PKR is best known for its role in virus infection and regulating cellular apoptosis [33,34]. In our model, simulating a down-regulation in the PKR node predicted a decrease in the LDH release (Table 2) and an increase in the Bcl-2 level in the palmitate cultures (Table 3). Indeed we found that inhibiting PKR (6 μM PKR inhibitor) in the palmitate cultures up-regulated the Bcl-2 protein expression (Figure 7B) and decreased the LDH release from ~50% to ~40%. Therefore, the model appropriately identified PKR to be an important factor involved in regulating Bcl-2 protein expression, and in turn the cytotoxicity.

**Table 3:** Simulating down-regulation of PKR and its effects on Bcl-2

		**Probability of Bcl-2 level**	
		
**Gene**	**level**	**Low**	**High**
	high	0.55	0.45
PKR	low	0.44	0.56 ↑

The probability of Bcl-2 taking on a high or low level in the palmitate cultures. BN inference was used to conduct the simulations of down-regulation of PKR. "↓" indicates decreased probability and "↑" indicates increased probability. This was validated experimentally as shown in Figure 7.

PKR was also found to be connected to apoptosis inhibitor (API5), which is connected to PP2AB56 and apoptotic chromatin condensation inducer (ACINUS), and the latter is connected to eIF2B suggesting that these factors are likely to be involved in the apoptotic signaling pathway. These connections are supported by published results in the literature. Caspase-3 activity was enhanced in the palmitate cultured cells (Figure 3), which may result in enhanced phosphorylation of eIF2-α. PKR can be cleaved by caspase-3, 7, 8 to liberate the eIF2-α kinase domain, which phosphorylates eIF2-α [35]. Phosphorylation of eIF2-α by PKR will inhibit protein synthesis and lead to apoptosis [35]. PKR also can bind to PP2A at the B56 alpha regulatory subunit (PP2AB56) and increase the phosphatase activity of PP2A [36]. PP2A is a major Ser/Thr phosphatase involved in many signal transduction pathways. PP2A can dephosphorylate and inactivate the anti-apoptotic Bcl-2 at Ser-70 [37].

#### 3.4 Activation of NF-κB by TNF-α is mediated by PKC-δ

**Figure 8:**
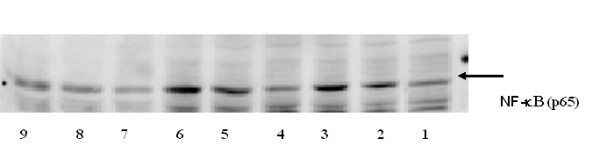
**Phosphorylated p65 subunit of NF-kB was determined by western blot with a monoclonal antibody**. 1) HepG2, 2) TNF-α at 20 ng/m; 3) TNFα at 100 ng/ml; 4) BSA 5) BSA+TNF-α at 20 ng/ml; 6) BSA+TNF-α at 100 ng/ml; 7) palmitate; 8) palmitate + TNF-α at 20 ng/ml; 9) palmitate + TNF-α at 100 ng/ml.

**Figure 9:**
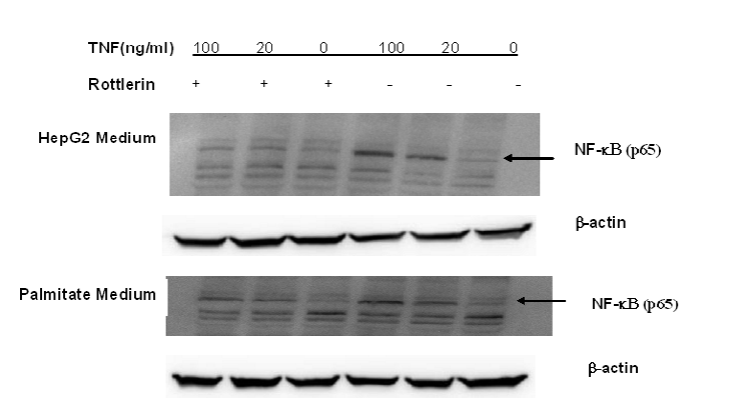
**Effect of rottlerin on NF-κB measured by western blotting**. Expression of phospho-P65 NF-κB in control and palmitate mediums with 0, 20, 100 ng/ml TNF-α, gith and without rottlerin (5 μM). TNF-α activated phospho-P65 NF-κB and this activation was attenuated with the PKC-δ inhibitor, rottlerin.

We found that the phospho-p65 NF-κB levels to be significantly lower in the palmitate cultures than in the oleate and linoleate (not shown) and control cultures shown in Figure 8. BN inference predicted that an up-regulation of NF-κB in the palmitate cultures would decrease the probability of LDH release being high (see Table 2). NF-κB is an important cytoprotective transcription factor, which can be activated by oxidative stress and cytokines, including TNF-α[38]. From Figure 4 we find that the connection between TNF-α and NF-κB is linked through PKC-δ, suggesting that PKC-δ is an intermediate factor in the activation of NF-κ B. Connections between TNF-α, PKC-δ and NF-κB have been identified in cells such as neutrophils [39] and pancreatic acinar cells [40]. Inhibiting PKC-δ has been shown to attenuate TNF-α-mediated activation of the anti-apoptotic transcription factor NF-κB in adherent neutrophils [39], but showed no effect on NF-κB activation in cultured myometrial cells [41], thus suggesting the pathway is cell dependent. There has been no study to date indicating that PKC-δ mediates TNF-α 's activation of NF-κB in HepG2 cells. Our model suggests that down-regulating PKC-δ will decrease the probability of NF-κB taking on a high expression level in the medium (plus TNF-α) cultures (Table 4). To determine whether PKC-δ is involved in mediating the activation of NF-κB by TNF-α in HepG2 cells, we added rottlerin, an inhibitor of PKC-δ, and measured the activity levels of NF-κB by western blotting. Rottlerin is a PKC-δ specific inhibitor that inhibits the tyrosine phosphorylation of PKC-δ, which to our knowledge does not interfere with any of the components in the NF-κB activation pathway. The activity of NF-κB was measured by detecting the levels of phosphorylated NF-κB p65 at Ser-536 [42]. As shown in Figure 9, the activation of NF-κB p65 was attenuated by rottlerin. Therefore, PKC-δ was appropriately identified by the model to be an important factor in mediating the TNF-α signaling to NF-κB.

**Table 4:** Simulating down-regulation of PKC-δ and its effects on NF-κβ.

		**Probability of NF-κβ activation**	
		
**Gene**	**level**	**Low**	**High**
	high	0.39	0.61
PKC-δ	low	0.51	0.49 ↓

The probability of NF-κβ taking on a high or low level. The model predicts that a down-regulation in PKC-δ will decrease the probability of NF-κβ taking on a high value. This was validated experimentally and shown in Figure 9. "↓" indicates decreased probability and "↑" indicates increased probability.

## Discussion

With the availability of high dimensional biological data to characterize a cellular state, one of the challenges is the development of robust methods that can integrate various orthogonal datasets to identify the genes and pathways that induce a phenotype. The significance of the *TIPS*^© ^framework is its ability to extract relevant information, both known and unknown, from high dimensional data. The phenotypic profile was used to guide the information extraction process. Proteins relay information from the genes to execute biological functions, which define the cellular phenotype. Thus, the effects of regulation occurring at the protein level manifest themselves in the phenotype. This paper illustrates that uncovering this information at the protein (i.e., intermediate) level may be achieved by integrating phenotype and gene expression data.

A handful of the connections were selected to illustrate the effectiveness of the framework. The selection was not intended to be comprehensive or exclusive of other potentially valid connections. The connections selected for further analysis and discussion were based upon i) evidence in the literature that a potential relationship may exist, although it may not be known what the exact nature of the relationship is or its relevance to toxicity, and ii) whether materials, e.g. assays or antibodies, are available to allow us to evaluate the connections.

Currently, only one phenotypic profile, e.g., LDH release, was used to identify the active network perturbed by TNF-α and FFA exposure. Metabolic profiles, which characterize the cellular phenotype, may also be used as constraints. Incorporating more metabolic profiles would improve the characterization of the phenotype and in turn the network reconstruction and predictions. An approach to add more constraints would be to apply ICA or PLS to extract several latent variables from the metabolic profiles [43]. In addition, the current *TIPS*^© ^approach selects genes based upon the data without considering a priori knowledge of the system under investigation. Using a purely data driven approach may result in important genes with modest changes escaping detection by statistical analysis such as ANOVA. Incorporating domain knowledge into the *TIPS*^© ^approach could improve the selection of relevant genes. The *prior *knowledge could be incorporated with approaches such as gene set enrichment analysis (GSEA) [44]. GSEA incorporates functional pathway information in the selection of significantly enriched functional gene groups.

Additionally, the current *TIPS*^© ^approach reconstructs pathways from gene expression and metabolic profiles at a single time point. Dynamic regulatory interactions may be inferred if data from multiple time points are available. Indeed, the underlying biological regulatory mechanism is likely to be dynamic in response to changes in the environmental conditions. A power law model has been applied in continuous dynamic Bayesian network (DBN) analysis to model the connection between genes [45,46]. The power law model can easily be extended to allow for delayed transcription [45,46]. Both discrete [47] and continuous [45,46] DBN has been applied to model gene regulatory networks. Therefore, to detect the dynamic property of biological networks, we plan to obtain time series data and incorporate a dynamic Bayesian network reconstruction component into the *TIPS*^© ^framework.

Due to computational limitation as well as limited data, it is not possible to reconstruct a network with high confidence using all the genes across the genome. GA/PLS and ICA provided an approach to identify a relevant subset of genes for further analysis. However, useful information may be missed in the selection process or not identified due to low abundance transcripts. To address the former, an approach would be to use a more targeted array with a smaller subset of genes. To address the latter, methods such as kinetically monitored reverse transcriptase-initiated PCR (kRT-PCR) could be used to measure genes with low abundance transcripts [48].

## Conclusion

In conclusion, we have demonstrated that *TIPS*^© ^may be applied to reconstruct the active associations from gene expression and phenotypic profiles to help elucidate the pathways involved in regulating palmitate-induced cytotoxicity. The pathways identified and shown in Figure 4 are specific to the cytotoxicity induced by FFA and TNF-α. If other compounds were applied to a cell culture system (HepG2 or another cell type), new microarray and phenotype data would have to be collected and the *TIPS*^© ^analysis applied to identify a different set of relevant genes specific to those compounds. This is important because many connections are context-specific (i.e. cell type and treatment). For instance, the regulation of Bcl-2 by TNFα is cell dependent. TNF-α suppresses Bcl-2 in FaO rat hepatoma cells [49] while it induces Bcl-2 in rat hippocampal neuron cells [50]. Similarly, signaling pathways are stimuli specific, for example, TNFα activates IKK activity with a negative feedback through A20 while LPS activates IKK activity with a positive feedback through a different pathway involving NF-κB and IRF3 [51]. Therefore, whether a pathway is activated will depend on the type of cell and the condition under consideration. The advantage of *TIPS*^© ^is that it allows for these differences and aims to reconstruct these pathways from the context-specific data.

## Methods

### Cell culture

One million Hep G2/C3A cells (ATCC, Manassa, VA) were seeded into each well of 6-well culture plate. The cells were kept in 2 ml of medium containing Dulbecco's modified Eagles medium (Invitrogen, Carlsbad, CA) supplemented with 10% fetal bovine serum (ATCC) and 2% Penicillin-streptomycin (Invitrogen). Cells were incubated at 37°C and in 10% CO2 atmosphere. After cells reached confluence, the medium was replaced with 2ml of treatment of FFA, either palmitate (700 μM), oleate (700 μM), or linoleate (700 μM), and in the presence and absence of TNF-α (0, 20, 100 ng/ml). Fatty-acid-free bovine serum albumin (BSA) was used as a carrier for the FFAs.

### Cytotoxicity measurement

For the LDH measurements, cells were cultured in different media for up to 48 hours and the supernatant collected. Cells were washed with phosphate buffered saline (PBS) and kept in 1% triton-X-100 in PBS for 24 h at 37°C. Cell lysate was then collected, vortexed for 15 seconds and centrifuged at 7000 rpm for 5 minutes. Cytotoxicity detection kit (Roche Applied Science, Indianapolis, IN) was used to measure the LDH release. LDH released was normalized to the total LDH (released + lyzed).

### Gene expression profiling

Cells were cultured in 10 cm tissue culture plates until confluence and then exposed to different treatments. RNA was isolated with Trizol reagent. The gene expression profiles were obtained with cDNA microarray. Analyses were done at the Van Andel Institute, Grand Rapids, MI. The protocols are available online at [52]. There were two biological replicates for each condition and each replicate was labeled with the Cy3 and Cy5 dyes. The microarray data has been deposited at the GEO website [53], with a query number of GSE5441.

### Inhibitors and activators

1 μM Rottlerin (inhibits PKC-δ, BIOMOL Research Laboratories, Plymouth Meeting, PA), 10 μM HA14-1 (inhibits Bcl-2, BIOMOL Research Laboratories, Plymouth Meeting, PA), 6 μM PKR inhibitor (Calbiochem, EMD Bioscience, CA) were used in the inhibitor experiments. 50 μM of clofibrate and ciprofibrate (Sigma) were used as activators of SCD.

### Immunoblotting

Cells were lysed with 1× SDS sample buffer. Proteins in cell lysates were separated on SDS-polyacrylamide gel electrophoresis (SDS-PAGE) and blotted onto nitrocellulose membrane. Non-specific binding sites were blocked with 5% non-fat milk. The membrane were then treated with anti-SCD (1:1000, Santa-Cruz Biotechnology Inc., CA), anti-PKC-δ (1:1000, Santa-Cruz Biotechnology Inc., CA), anti-Bcl-2 (1:2000, Cell signaling Inc., MA), and anti-NF-κB-P65 (1:1000, Cell signaling Inc., MA) to detect protein level of SCD, PKC-δ, Bcl-2, and NF-κB, respectively. Membranes were also immnoblotted with anti-β-actin (1:1000, Cell signaling Inc., MA) to monitor the loading level in each lane. All antibodies are against the human isoform.

### Data analysis

#### ANOVA

The analysis of variance (ANOVA) was applied to compare the effect of treatment (e.g. FFA, TNF-α) and to determine whether a treatment has a significant effect. We applied a two-way ANOVA test to identify the genes that are affected by FFA, TNF-α or the interaction between FFA and TNF-α. The analysis was performed in MATLAB 6.3 using Stats Toolbox. A two step ANOVA analysis was performed to identify the genes that changed significantly due to FFA or TNF-α exposure. We identified a list of genes from the literature shown in the additional files [see Additional file 2], that are relevant to palmitate-induced cytotoxicity and applied ANOVA with P ≤ 0.05 to this list of genes (which we denote as "supervised" ANOVA). In addition, ANOVA analysis was applied to the entire list of genes with P ≤ 0.01 (which we denote as "unsupervised" ANOVA). The two lists of genes were then combined into one list, eliminating any overlaps between the lists. Using the supervised and unsupervised ANOVA tests, the expression level of 830 genes were found to be significant due to either TNF-α or FFA. This list of genes is in the additional files [see Additional file 3].

#### TIPS^© ^framework

**Figure 10:**
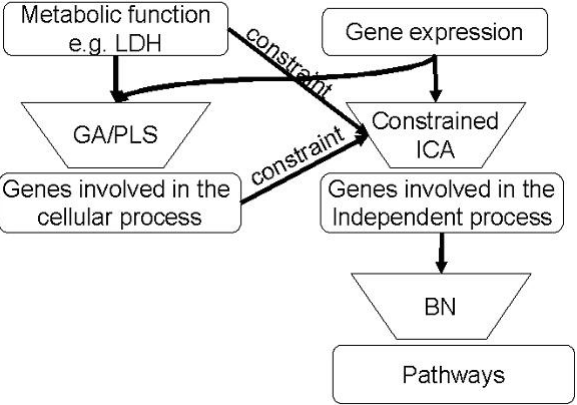
**The framework to integrate gene expression and metabolic profiles**. The relevancy of each gene to LDH release was first evaluated with GA/PLS. Genes with higher frequencies were considered to be more relevant. The frequencies and profile of the LDH release were then used to constrain the ICA model to extract an independent component that represents the cellular process. The genes in the independent component with high coefficients were subjected to BN analysis.

We apply a mathematical framework that first integrates genetic algorithm (GA) and partial least squares (PLS) analyses to identify the genes relevant to LDH release, but these genes may be involved in many independent pathways. Therefore, the framework then applies CICA to identify an independent pathway involved in LDH release. Finally the connections between these genes selected by CICA are reconstructed using BN analysis to infer how the genes interact with each other in the independent pathways. The reconstructed network illustrates how the genes interact under the given environmental conditions to regulate LDH release. The framework is shown schematically in Figure 10.

#### Genetic algorithm/partial least square analysis (GA/PLS)

Based upon the notion that metabolic functions are regulated in part by the enzymes catalyzing the reactions, which in turn are determined in part by their gene expression levels, we hypothesize that the metabolic function can be predicted from the expression level of a subset of genes that are associated with the metabolic function. The log-linear model (also known as the power law function) was used to approximate the non-linear relation between metabolic function and gene expression levels. Log-liner model has its roots in biochemistry and has been applied by Savageau et. al. (also coined as S-system) to approximate the relation between reaction rates and their substrates [54-56]. The log-linear model has also been applied successfully to model the expression of a gene as a power law function of the expression of the genes that regulate it [45]. The advantage of log-linear models is that they are computationally tractable and robust [56] and restricted nonlinear relationships have been modeled as well. Although, mechanism based nonlinear models [57] can capture more accurate behavior, they require more parameters and more data to estimate these parameters and in turn higher computational cost. Therefore, we used the log-linear model to approximate the non-linear relations between phenotype and gene expression level shown in equation (1).

Met(treatment)Met(control)=∏i=1n(Gene(treatment)iGene(control)i)C(i)
 MathType@MTEF@5@5@+=feaafiart1ev1aaatCvAUfKttLearuWrP9MDH5MBPbIqV92AaeXatLxBI9gBaebbnrfifHhDYfgasaacH8akY=wiFfYdH8Gipec8Eeeu0xXdbba9frFj0=OqFfea0dXdd9vqai=hGuQ8kuc9pgc9s8qqaq=dirpe0xb9q8qiLsFr0=vr0=vr0dc8meaabaqaciaacaGaaeqabaqabeGadaaakeaadaWcaaqaaiabd2eanjabdwgaLjabdsha0jabcIcaOiabdsha0jabdkhaYjabdwgaLjabdggaHjabdsha0jabd2gaTjabdwgaLjabd6gaUjabdsha0jabcMcaPaqaaiabd2eanjabdwgaLjabdsha0jabcIcaOiabdogaJjabd+gaVjabd6gaUjabdsha0jabdkhaYjabd+gaVjabdYgaSjabcMcaPaaacqGH9aqpdaqeWbqaaiabcIcaOmaalaaabaGaem4raCKaemyzauMaemOBa4MaemyzauMaeiikaGIaemiDaqNaemOCaiNaemyzauMaemyyaeMaemiDaqNaemyBa0MaemyzauMaemOBa4MaemiDaqNaeiykaKYaaSbaaSqaaiabdMgaPbqabaaakeaacqWGhbWrcqWGLbqzcqWGUbGBcqWGLbqzcqGGOaakcqWGJbWycqWGVbWBcqWGUbGBcqWG0baDcqWGYbGCcqWGVbWBcqWGSbaBcqGGPaqkdaWgaaWcbaGaemyAaKgabeaaaaaabaGaemyAaKMaeyypa0JaeGymaedabaGaemOBa4ganiabg+GivdGccqGGPaqkdaahaaqabeaacqWGdbWqcqGGOaakcqWGPbqAcqGGPaqkaaaaaa@830E@

where *Met(treatment) *and *Met(control) *are the metabolic function for the treated and control cultures, respectively; *Gene(treatment)*_*i *_and *Gene(control)*_*i *_are the expression level of gene *i *for the treated and control cultures, respectively.

Denoting Y as log⁡2(Met(treatment)Met(control))
 MathType@MTEF@5@5@+=feaafiart1ev1aaatCvAUfKttLearuWrP9MDH5MBPbIqV92AaeXatLxBI9gBaebbnrfifHhDYfgasaacH8akY=wiFfYdH8Gipec8Eeeu0xXdbba9frFj0=OqFfea0dXdd9vqai=hGuQ8kuc9pgc9s8qqaq=dirpe0xb9q8qiLsFr0=vr0=vr0dc8meaabaqaciaacaGaaeqabaqabeGadaaakeaacyGGSbaBcqGGVbWBcqGGNbWzdaWgaaWcbaGaeGOmaidabeaakiabcIcaOmaalaaabaGaemyta0KaemyzauMaemiDaqNaeiikaGIaemiDaqNaemOCaiNaemyzauMaemyyaeMaemiDaqNaemyBa0MaemyzauMaemOBa4MaemiDaqNaeiykaKcabaGaemyta0KaemyzauMaemiDaqNaeiikaGIaem4yamMaem4Ba8MaemOBa4MaemiDaqNaemOCaiNaem4Ba8MaemiBaWMaeiykaKcaaiabcMcaPaaa@5520@ and X_*i *_as log⁡2(Gene(treatment)iGene(control)i)
 MathType@MTEF@5@5@+=feaafiart1ev1aaatCvAUfKttLearuWrP9MDH5MBPbIqV92AaeXatLxBI9gBaebbnrfifHhDYfgasaacH8akY=wiFfYdH8Gipec8Eeeu0xXdbba9frFj0=OqFfea0dXdd9vqai=hGuQ8kuc9pgc9s8qqaq=dirpe0xb9q8qiLsFr0=vr0=vr0dc8meaabaqaciaacaGaaeqabaqabeGadaaakeaacyGGSbaBcqGGVbWBcqGGNbWzdaWgaaWcbaGaeGOmaidabeaakiabcIcaOmaalaaabaGaem4raCKaemyzauMaemOBa4MaemyzauMaeiikaGIaemiDaqNaemOCaiNaemyzauMaemyyaeMaemiDaqNaemyBa0MaemyzauMaemOBa4MaemiDaqNaeiykaKYaaSbaaSqaaiabdMgaPbqabaaakeaacqWGhbWrcqWGLbqzcqWGUbGBcqWGLbqzcqGGOaakcqWGJbWycqWGVbWBcqWGUbGBcqWG0baDcqWGYbGCcqWGVbWBcqWGSbaBcqGGPaqkdaWgaaWcbaGaemyAaKgabeaaaaGccqGGPaqkaaa@5AB8@, equation (1) is transformed to a log-linear model:

Y=∑i=1nC(i)Xi
 MathType@MTEF@5@5@+=feaafiart1ev1aaatCvAUfKttLearuWrP9MDH5MBPbIqV92AaeXatLxBI9gBaebbnrfifHhDYfgasaacH8akY=wiFfYdH8Gipec8Eeeu0xXdbba9frFj0=OqFfea0dXdd9vqai=hGuQ8kuc9pgc9s8qqaq=dirpe0xb9q8qiLsFr0=vr0=vr0dc8meaabaqaciaacaGaaeqabaqabeGadaaakeaacqWGzbqwcqGH9aqpdaaeWbqaaiabdoeadjabcIcaOiabdMgaPjabcMcaPaWcbaGaemyAaKMaeyypa0JaeGymaedabaGaemOBa4ganiabggHiLdGccqWGybawdaWgaaWcbaGaemyAaKgabeaaaaa@3CCB@

In this study the coefficients *C(i) *in equation (2) are determined by PLS analysis and the genes, *Gene*_*i*_, are selected by GA/PLS as described in reference [9].

#### Constrained Independent Component Analysis (CICA)

ICA is a statistical method that has been applied to reveal "hidden factors" underlying sets of signal measurements [10]. The expression levels of the genes are the recorded signals which are affected by underlying regulatory pathways. We denote the signal measurements **Y **to be the gene expression data, **S **to be the independent components (pathways), and **A **to be the mixing matrix. The ICA model can be expressed as

**Y = A S**

The gene expression **Y **is supplied to the ICA model and the mixing matrix **A **can be uniquely estimated by assuming that the components in **S **are statistically independent to each other. *Y(i,t) *represents the expression of gene *i *in experiment *t*, *A(i,j) *represents weight of gene *i *in independent pathway *j, S(j,t) *represents the profile of independent pathway *j *in experiment *t*. Since the objective here is to identify a LDH release related independent pathway, **A **was further constrained with the frequency learned by GA/PLS and **S **was further constrained with the LDH release profile. Let *F(i) *be the frequency of gene *i *with respect to LDH release and ***a**(i) *be the weight of gene *i *in the pathway related to LDH release. Thus, ***a ***is constrained to have a correlation with *F *by equation (4), where ρ1 is a threshold value.

Corr1 = a^T ^*diag (F)*a/(a^T ^*a) > ρ1

Let *s(t) *be the profile of LDH related pathway in experiment *t *and *L(t) *be the profile of LDH release in experiment *t*. Similarly, ***s ***is constrained to have a correlation with *L *by equation (5), where ρ2 is a threshold value.

Corr2 = s^T^*L*L^T^*s/(s*s^T^) >ρ2

Implicit in the use of ICA is i) ICA separates only linear effects, ii) all the genes are assumed to be linearly independent, and iii) can handle only one Gaussian source, which is typically assumed to be the noise in the data, and the independent signal sources are non-Gaussian. The assumption that information flow from genes to proteins is linear is a reasonable first-order approximation since most genes translate to proteins which are not involved in regulation or feedback loops. We addressed the second assumption through pre-whitening the gene expression data with singular value decomposition to remove the principal components with small eigenvalues, i.e. linear dependency. For the third assumption, we evaluated the normality of the independent component and found the extracted independent component is non-Gausssian [see Additional file 4], therefore justifying the application of ICA to the gene data.

### Bayesian network analysis

Bayesian networks are directed acyclic graphs (DAG) whose nodes correspond to variables and whose arcs represent the dependencies between variables. The dependencies are determined by the conditional probabilities of each node *x*_*i*_, given its parent node *p*_*a*_, Pr(*x*_*i *_| *p*_*a*_(*x*_*i*_)). A Bayesian network i) assumes conditional independence, such that each node is independent to its non-descendants, given it parents. For example, *B *and *C *are conditionally independent to each other given *A *in a network, then

*Pr*(*B *| *C,A*) = *Pr*(*B*|*A*)

and ii) consists of joint distribution defined by a set of variables {*x*_i_} as:

Pr⁡(x1,....,xn)=∏i=1NPr⁡(xi|pa(xi))
 MathType@MTEF@5@5@+=feaafiart1ev1aaatCvAUfKttLearuWrP9MDH5MBPbIqV92AaeXatLxBI9gBaebbnrfifHhDYfgasaacH8akY=wiFfYdH8Gipec8Eeeu0xXdbba9frFj0=OqFfea0dXdd9vqai=hGuQ8kuc9pgc9s8qqaq=dirpe0xb9q8qiLsFr0=vr0=vr0dc8meaabaqaciaacaGaaeqabaqabeGadaaakeaacyGGqbaucqGGYbGCcqGGOaakcqWG4baEdaWgaaWcbaGaeGymaedabeaakiabcYcaSiabc6caUiabc6caUiabc6caUiabc6caUiabcYcaSiabdIha4naaBaaaleaacqWGUbGBaeqaaOGaeiykaKIaeyypa0ZaaebCaeaacyGGqbaucqGGYbGCcqGGOaakcqWG4baEdaWgaaWcbaGaemyAaKgabeaakiabcYha8jabdchaWnaaBaaaleaacqWGHbqyaeqaaOGaeiikaGIaemiEaG3aaSbaaSqaaiabdMgaPbqabaGccqGGPaqkcqGGPaqkaSqaaiabdMgaPjabg2da9iabigdaXaqaaiabd6eaobqdcqGHpis1aaaa@541C@

In the example above:

*Pr *(*A*, *B*, *C*) = *Pr *(*A*) *Pr *(*B *| *A*) *Pr *(*C *| *A*)

There are two common approaches that have been applied to learn the network structure, the score and search based and the constraint based methods. This study compared the score and search based LibB [6] method with the constraint based greedy thickening and thinning algorithm [58]. The score and search based LibB method, downloaded from Nir Friedman's group at [59], used the sparse candidate algorithm to search for a network with the highest score [6]. The score S(G:D) is defined to be proportional to P(G|D) and is used to evaluate the networks, where G represents the graph and D represents the training dataset and P(G|D) represent probability of Graph G given data D. S(G:D) can be decomposed according to equation (9).

S(G:D)=∑iSlocal(Xi,PaiG:D)
 MathType@MTEF@5@5@+=feaafiart1ev1aaatCvAUfKttLearuWrP9MDH5MBPbIqV92AaeXatLxBI9gBaebbnrfifHhDYfgasaacH8akY=wiFfYdH8Gipec8Eeeu0xXdbba9frFj0=OqFfea0dXdd9vqai=hGuQ8kuc9pgc9s8qqaq=dirpe0xb9q8qiLsFr0=vr0=vr0dc8meaabaqaciaacaGaaeqabaqabeGadaaakeaacqWGtbWucqGGOaakcqWGhbWrcqGG6aGocqWGebarcqGGPaqkcqGH9aqpdaaeqbqaaiabdofatnaaBaaaleaacqWGSbaBcqWGVbWBcqWGJbWycqWGHbqycqWGSbaBaeqaaOGaeiikaGIaemiwaG1aaSbaaSqaaiabdMgaPbqabaGccqGGSaalcqWGqbaucqWGHbqydaqhaaWcbaGaemyAaKgabaGaem4raCeaaOGaeiOoaOJaemiraqKaeiykaKcaleaacqWGPbqAaeqaniabggHiLdaaaa@4BE3@

Slocal(X,iU:D)=log⁡P(Pai=U)+log⁡∫∏mP(Xi[m]|U[m],θ)dP(θ)
 MathType@MTEF@5@5@+=feaafiart1ev1aaatCvAUfKttLearuWrP9MDH5MBPbIqV92AaeXatLxBI9gBaebbnrfifHhDYfgasaacH8akY=wiFfYdH8Gipec8Eeeu0xXdbba9frFj0=OqFfea0dXdd9vqai=hGuQ8kuc9pgc9s8qqaq=dirpe0xb9q8qiLsFr0=vr0=vr0dc8meaabaqaciaacaGaaeqabaqabeGadaaakeaacqWGtbWudaWgaaWcbaGaemiBaWMaem4Ba8Maem4yamMaemyyaeMaemiBaWgabeaakiabcIcaOiabdIfaynaaBeaaleaacqWGPbqAaeqaaOGaeiilaWIaemyvauLaeiOoaOJaemiraqKaeiykaKIaeyypa0JagiiBaWMaei4Ba8Maei4zaCMaemiuaaLaeiikaGIaemiuaaLaemyyae2aaSbaaSqaaiabdMgaPbqabaGccqGH9aqpcqWGvbqvcqGGPaqkcqGHRaWkcyGGSbaBcqGGVbWBcqGGNbWzdaWdbaqaamaarafabaGaemiuaaLaeiikaGIaemiwaG1aaSbaaSqaaiabdMgaPbqabaGccqGGBbWwcqWGTbqBcqGGDbqxcqGG8baFcqWGvbqvcqGGBbWwcqWGTbqBcqGGDbqxcqGGSaaliiGacqWF4oqCcqGGPaqkcqWGKbazcqWGqbaucqGGOaakcqWF4oqCcqGGPaqkaSqaaiabd2gaTbqab0Gaey4dIunaaSqabeqaniabgUIiYdaaaa@6EB6@

The first term in equation (10), log *P *(*Pa*_*i *_= *U*), is the prior probability for the choice of U as the parent of X, the second term in equation (10) calculates the probability of the data given the possible values for the parameter, θ; θ defines the conditional probabilities between the nodes. The score S(G:D) is maximized using the sparse candidate algorithm in the structure learning process. Mutual information based algorithm was used for the constraint based network reconstruction, the details of the algorithm can be found in [4,58] and consists of 3 phases. Briefly, in Phase 1 the mutual information contained in each pair of genes is calculated as a measure of closeness, indicating the correlation between the pair of genes. The algorithm creates a draft of the regulatory network based upon the calculated mutual information. The mutual information *I(X*_*i*_*,X*_*j*_) for each pair of nodes (*x*_*i*_*,x*_*j*_) is computed as follows:

I(Xi,Xj)=∑xi,xjPr⁡(xi,xj)log⁡Pr⁡(xi,xj)Pr⁡(xi)P(xj)
 MathType@MTEF@5@5@+=feaafiart1ev1aaatCvAUfKttLearuWrP9MDH5MBPbIqV92AaeXatLxBI9gBaebbnrfifHhDYfgasaacH8akY=wiFfYdH8Gipec8Eeeu0xXdbba9frFj0=OqFfea0dXdd9vqai=hGuQ8kuc9pgc9s8qqaq=dirpe0xb9q8qiLsFr0=vr0=vr0dc8meaabaqaciaacaGaaeqabaqabeGadaaakeaacqWGjbqscqGGOaakcqWGybawdaWgaaWcbaGaemyAaKgabeaakiabcYcaSiabdIfaynaaBaaaleaacqWGQbGAaeqaaOGaeiykaKIaeyypa0ZaaabuaeaacyGGqbaucqGGYbGCcqGGOaakcqWG4baEdaWgaaWcbaGaemyAaKgabeaakiabcYcaSiabdIha4naaBaaaleaacqWGQbGAaeqaaOGaeiykaKIagiiBaWMaei4Ba8Maei4zaC2aaSaaaeaacyGGqbaucqGGYbGCcqGGOaakcqWG4baEdaWgaaWcbaGaemyAaKgabeaakiabcYcaSiabdIha4naaBaaaleaacqWGQbGAaeqaaOGaeiykaKcabaGagiiuaaLaeiOCaiNaeiikaGIaemiEaG3aaSbaaSqaaiabdMgaPbqabaGccqGGPaqkcqWGqbaucqGGOaakcqWG4baEdaWgaaWcbaGaemOAaOgabeaakiabcMcaPaaaaSqaaiabdIha4naaBaaameaacqWGPbqAaeqaaSGaeiilaWIaemiEaG3aaSbaaWqaaiabdQgaQbqabaaaleqaniabggHiLdaaaa@67F5@

Then each pair of genes with mutual information *I(X*_*i*_*,X*_*j*_) greater than a threshold value, **T**, are sorted in a list L from high to low. **T **is set to be the default value in BN PowerConstructor. An arc is drawn for the first two pairs of nodes in L. The pointer is then moved to the next pair of nodes. If no path exists between the pair an arc is added. In Phase 2 arcs are added when pairs of unconnected nodes are dependent as determined by the conditional independence (CI) test. The CI test is based on conditional mutual information as defined by equation (12) below. For *I(Xi,Xj|c) *less than the specified threshold value **T**, (*Xi,Xj*) is said to be independent given **c**, where **c **is a set of genes.

I(Xi,Xj|c)=∑xi,xj,cPr⁡(xi,xj,c)log⁡Pr⁡(xi,xj|c)Pr⁡(xi|c)P(xj|c)
 MathType@MTEF@5@5@+=feaafiart1ev1aaatCvAUfKttLearuWrP9MDH5MBPbIqV92AaeXatLxBI9gBaebbnrfifHhDYfgasaacH8akY=wiFfYdH8Gipec8Eeeu0xXdbba9frFj0=OqFfea0dXdd9vqai=hGuQ8kuc9pgc9s8qqaq=dirpe0xb9q8qiLsFr0=vr0=vr0dc8meaabaqaciaacaGaaeqabaqabeGadaaakeaacqWGjbqscqGGOaakcqWGybawdaWgaaWcbaGaemyAaKgabeaakiabcYcaSiabdIfaynaaBaaaleaacqWGQbGAaeqaaOGaeiiFaWNaem4yamMaeiykaKIaeyypa0ZaaabuaeaacyGGqbaucqGGYbGCcqGGOaakcqWG4baEdaWgaaWcbaGaemyAaKgabeaakiabcYcaSiabdIha4naaBaaaleaacqWGQbGAaeqaaOGaeiilaWIaem4yamMaeiykaKIagiiBaWMaei4Ba8Maei4zaC2aaSaaaeaacyGGqbaucqGGYbGCcqGGOaakcqWG4baEdaWgaaWcbaGaemyAaKgabeaakiabcYcaSiabdIha4naaBaaaleaacqWGQbGAaeqaaOGaeiiFaWNaem4yamMaeiykaKcabaGagiiuaaLaeiOCaiNaeiikaGIaemiEaG3aaSbaaSqaaiabdMgaPbqabaGccqGG8baFcqWGJbWycqGGPaqkcqWGqbaucqGGOaakcqWG4baEdaWgaaWcbaGaemOAaOgabeaakiabcYha8jabdogaJjabcMcaPaaaaSqaaiabdIha4naaBaaameaacqWGPbqAaeqaaSGaeiilaWIaemiEaG3aaSbaaWqaaiabdQgaQbqabaWccqGGSaalcqWGJbWyaeqaniabggHiLdaaaa@778F@

In Phase 3 each arc is examined using the CI test and arcs are removed if two genes linked by an arc are conditionally independent. The LDH node was assigned as a leaf node in the learning process, which enabled the determination of direction in the network. BN PowerConstructor downloaded from Cheng Jie's group at [60] was used in the constraint based network reconstruction. Both the score and search based and the constraint based approaches have their advantages and disadvantages in different respects. For example, Heckerman *et al*. [61] showed that the scoring-based method is advantageous over the constraint-based methods when modeling a probability distribution of observations. However, Friedman *et al*. [62] showed theoretically that the general scoring-based method may result in poor prediction accuracy, which was also confirmed by Greiner *et al*. [63]. Score and search method often are computationally more costly [61]. Constraint based method, on the other hand, is able to discriminate between models with and without hidden variables and can indicate the presence of a hidden common cause between two variables [64]. Therefore, we applied both approaches and compared the network learned using our data. We found that fewer connections were identified using the LibB approach and the LDH node was not connected to any genes (the full network is shown in the additional files [see Additional file 5]). Based upon this comparison, we decided to use the constraint based approach to generate the network for subsequent analyses.

### Bayesian network inference

Bayesian network inference was used to predict the probabilities of a phenotype e.g. LDH or a gene will take a certain value with the other genes in the network at controlled levels. The posterior probability that the class node will take on a certain value given the values of the other nodes is determined based upon conditional probability. Suppose node A is the target node and b1 and c1 are the known values of evidence nodes B and C, respectively, we can predict the posterior probability Pr(A| xi, xj) according to the Bayesian rule:

Pr(A=a_1_|B=b_1, _C=c_1_)=Pr(B=b_1_,C=c_1_|A=a_1_)*Pr(A=a_1_)/Pr(B=b_1_,C=c_1_) (9)

However, applying this exact inference to a large network is computationally costly [65]. Therefore, we applied an approximate inference algorithm, logic sampling [66], to infer the posterior probabilities. Briefly, logic sampling generates a case by randomly assigning values to each node weighted by the probability of that value occurring. To estimate the posterior probability Pr(X|E) where X is target node and E is the evidence node, we compute the ratio of the number of cases where both E and X are true to the number of cases where just E is true, i.e. Pr(X=x|E=e) = Pr(X=x, E=e)/Pr(E=e). The inference process was conducted with Genie [67].

## Availability and requirements

Project name: Three Stage Integrative Pathway Search (*TIPS^©^*)

Project home page: www.egr.msu.edu/tips

Operating system(s): Platform independent

Programming language: Matlab

License: GNU GPL for academics, license needed for non-academic users

## Abbreviations

ACC acetyl-CoA carboxylase

AMPK AMP-activated protein kinase

TIPS three-stage-integrative-pathway-search

FFA free fatty acid

GA/PLS genetic algorithm coupled partial least square analysis

ICA independent component analysis

LDH lactate dehydrogenase

NF-κB nuclear factor kappa B

PKC-δ protein kinase C delta

PKR double-stranded-RNA-dependent protein kinase

SCD stearoyl-CoA desaturase

TNF-α tumor necrosis factor alpha

## Authors' contributions

ZL conceived the methodology and performed part of the experiments and wrote the manuscript. SS performed the cell culture, LDH measurement, microarray measurement, caspase measurement, wrote part of the manuscript. SM performed cell culture and microarray measurement. XY performed PKR and Bcl-2 experiment and wrote part of the manuscript. LS performed western blot experiment. CC conceived the study and supervised the experiment and writing of the manuscript. All authors read and approved the final manuscript.

## Supplementary Material

Additional file 1 ICA gene weights. Additional file 1 is a list of the gene weights determined by ICA. Click here for file

Additional file 2 Important gene list. Additional file 2 is a list of important genes determined manually with a literature review. Click here for file

Additional file 3 Genes selected by ANOVA. Additional file 3 lists 830 genes selected by ANOVA analysis. Click here for file

Additional file 4 Normality test. Additional file 4 presents the normality test of the gene data. Click here for file

Additional file 5 Network learned by LibB. Additional file 5 presents the network learned by search and score method of LibB. Click here for file
