# Supplementary figures and images for: A Three Stage Integrative Pathway Search (TIPS©) framework to identify toxicity relevant genes and pathways
Source: BMC Bioinformatics. 2007 Jun 14;8:202. doi: 10.1186/1471-2105-8-202 (PMC1906836; doi:10.1186/1471-2105-8-202)

Normality test of the independent component


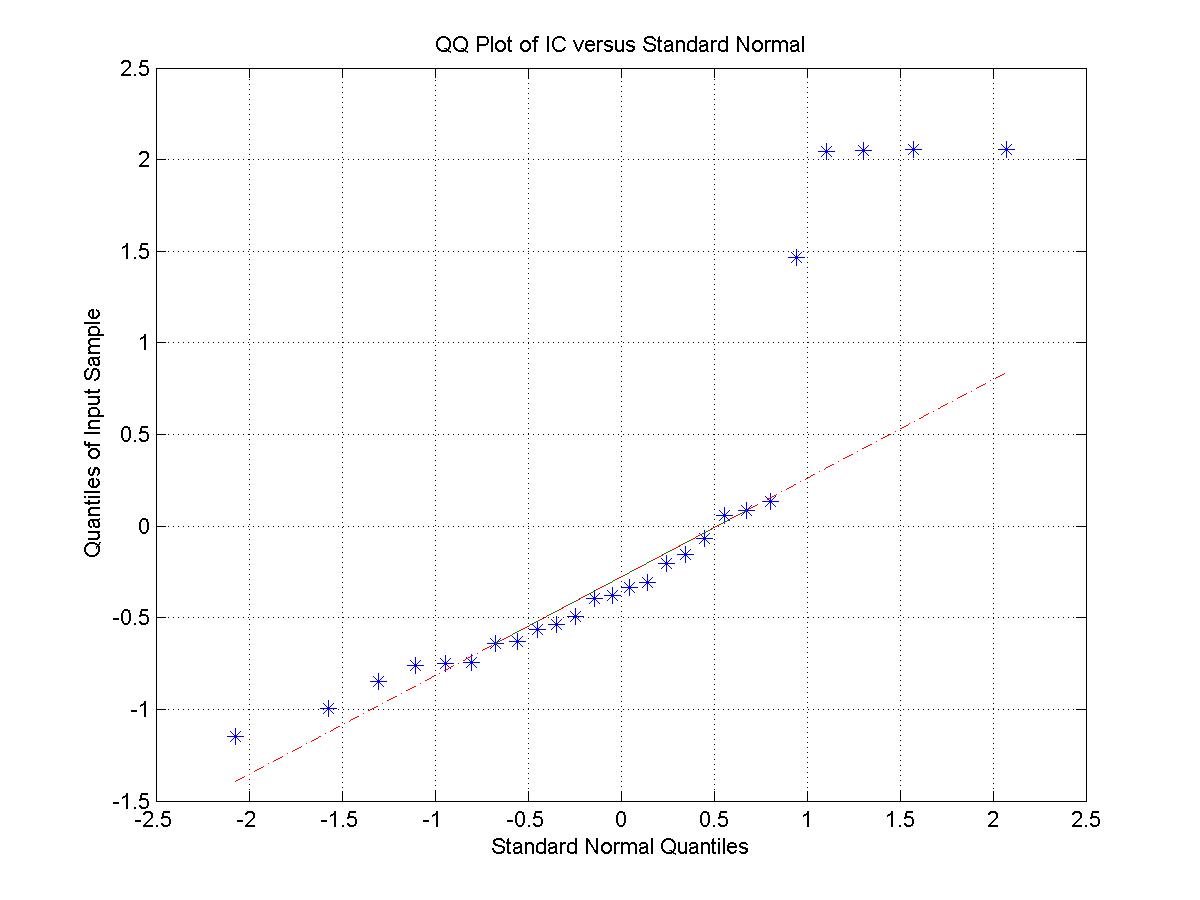

Supplement: Additional file 4 — Normality test. Additional file 4 presents the normality test of the gene data. [file 1471-2105-8-202-S4.doc]

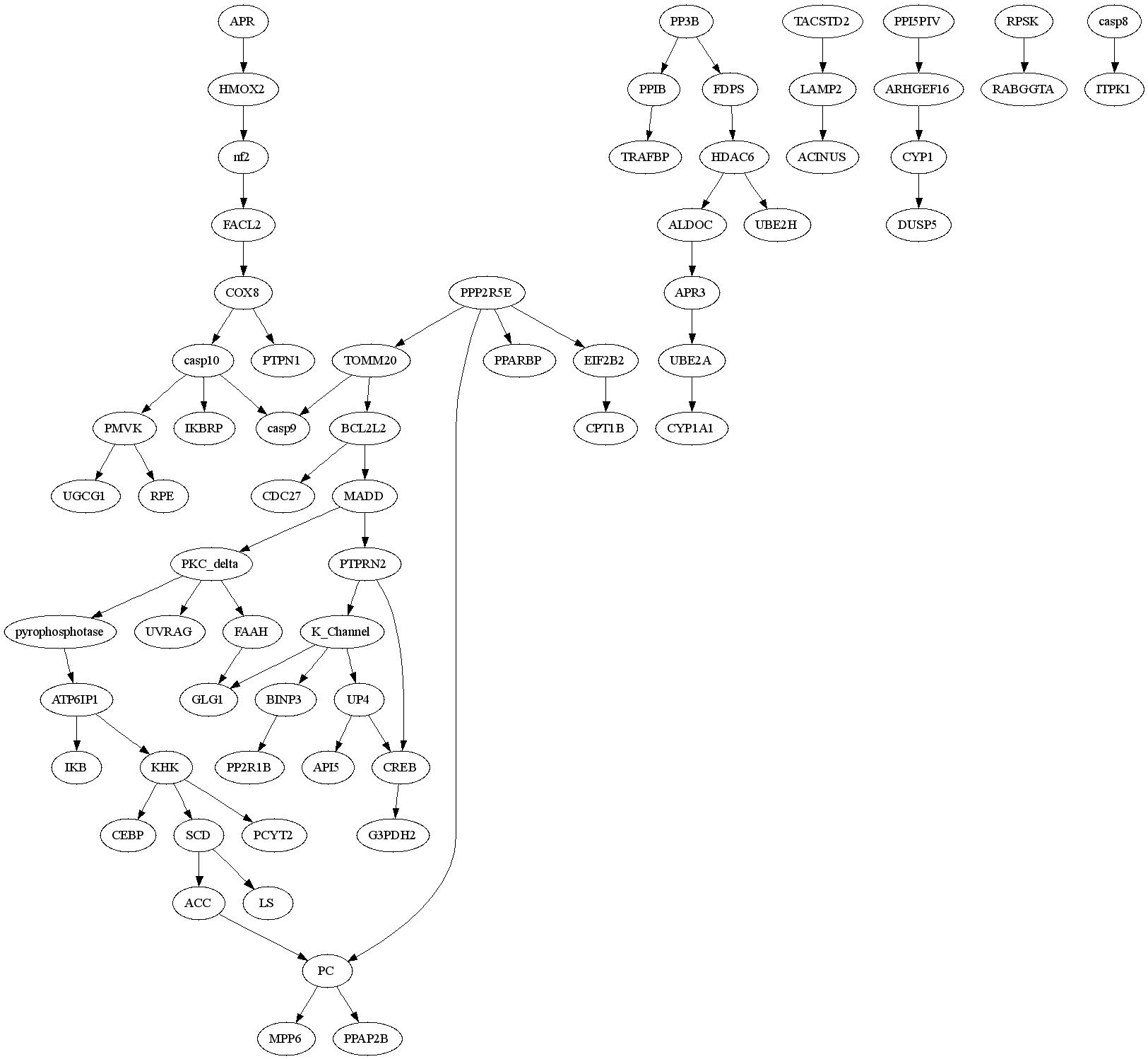

Supplement: Additional file 5 — Network learned by LibB. Additional file 5 presents the network learned by search and score method of LibB. [file 1471-2105-8-202-S5.jpeg]
